# Synthesis and structure of *catena*-poly[[[aquazinc(II)]-μ-2-({[amino­(sulfanid­yl)methyl­idene]amino}­imino)-2-phenyl­acetato-κ^4^*O*,*N*,*S*:*O*′] monohydrate]

**DOI:** 10.1107/S2056989026010169

**Published:** 2026-10-02

**Authors:** Javokhirkhon Akhatov, Gulnora Tillaeva, Khayit Turaev, Odyl Choriyev, Maftuna Primkulova, Bakhtiyar Ibragimov, Jamshid Mengnorovich Ashurov

**Affiliations:** a Termez State University, Barkamol Avlod St. 43, 190111, Termez, Uzbekistan; b Tashkent Pharmaceutical Institute A. Aybek St. 45 Tashkent 100015 Uzbekistan; c Institute of Bioorganic Chemistry, Academy of Sciences of Uzbekistan, 100125, M. Ulugbek Str 83, Tashkent, Uzbekistan; d Denov Institute of Entrepreneurship and Pedagogy Bog St. 112, Denov, 733500, Uzbekistan; Vienna University of Technology, Austria

**Keywords:** zinc(II), coordination polymer, benzketozone, phenyl­glyoxylic acid thio­semicarbazone, crystal structure, Hirshfeld surface analysis, hydrogen bonding

## Abstract

In the polymeric title compound, {[Zn(C_9_H_7_N_3_O_2_S)(H_2_O)]·H_2_O}_*n*_, the dianion of phenyl­glyoxylic acid thio­semicarbazone (benzketozone) chelates one Zn^II^ cation through carboxyl­ate O, azomethine N and thiol­ate S atoms and bridges to the next Zn^II^ ion through its second carboxyl­ate O atom. Each Zn^II^ ion is five-coordinate, with the aqua ligand completing the coordination sphere, which has a shape inter­mediate between square-pyramidal and trigonal-bipyramidal. The coordination motif generates a chain extending parallel to [010], with an adjacent Zn⋯Zn separation of 4.8378 (4) Å. O—H⋯O, O—H⋯S and N—H⋯N hydrogen bonds connect the polymeric chains and non-coordinating water mol­ecules into a three-periodic network; a weak N—H⋯π contact is also present. Hirshfeld surface analysis identifies H⋯H (34.8%), C⋯H/H⋯C (16.1%), H⋯S/S⋯H (13.2%), O⋯H/H⋯O (11.0%) and N⋯H/H⋯N (8.4%) as the principal contact contributions.

## Abstract 2

The title Zn^II^ compound is a mono-periodic coordination polymer in which the doubly deprotonated phenyl­glyoxylic acid thio­semicarbazone ligand chelates one Zn^II^ ion through O, N and S donors and bridges to the next through its second carboxyl­ate O atom.

## 1. Chemical context

Thio­semicarbazones are versatile Schiff-base ligands whose coordination behaviour is strongly influenced by the parent carbonyl compound, the central metal cation and the degree of ligand deprotonation. They commonly bind through the azomethine N and thione/thiol­ate S atoms, while additional donor groups can lead to tridentate or higher-denticity coordination modes (Casas *et al.*, 2000 ▸; Lobana *et al.*, 2009 ▸; Dilworth & Hueting, 2012 ▸). Their structural diversity, together with the broad biological activity of thio­semicarbazone derivatives and their metal complexes, continues to attract considerable inter­est (Zahra *et al.*, 2025 ▸). Anti­cancer, anti­microbial, anti­viral, anti­malarial and anti­oxidant activities have been reported, together with inter­actions involving albumin, DNA and biologically relevant enzymes (Devi *et al.*, 2024 ▸; Nascimento *et al.*, 2024 ▸; Chen *et al.*, 2025 ▸; Ucar *et al.*, 2025 ▸). Selected radiometal bis­(thio­semicarbazone) complexes have also been investigated for diagnostic PET/SPECT imaging and therapeutic applications (Dilworth & Hueting, 2012 ▸).

Thio­semicarbazones derived from α-keto carb­oxy­lic acids are particularly attractive because the carboxyl­ate group provides an additional O-donor site. Deprotonation of both the carboxyl group and the hydrazinic N atom thus can generate a dianionic O,N,S donor set, while the second carboxyl­ate O atom may participate in bridging, thereby allowing both discrete chelating modes and the formation of coordination polymers. Glyoxylic acid thio­semicarbazone (H_2_GAT) illustrates this structural diversity: Co^II^ and Ni^II^ form mononuclear octa­hedral bis-chelate complexes (Huseynova *et al.*, 2018 ▸a), Zn^II^ gives discrete complexes under some conditions (Huseynova *et al.*, 2020 ▸, 2022 ▸), whereas Cu^II^ and Zn^II^ can form one-periodic polymers through hydrazinic-N and carboxyl­ate-O bridging, respectively (Burshtein *et al.*, 1987 ▸; Huseynova *et al.*, 2019*a* ▸,*b* ▸). Related Zn^II^ coordination polymers have also been investigated using crystallographic, Hirshfeld surface and theoretical approaches (Rasulovich *et al.*, 2024 ▸; Nuralieva *et al.*, 2025 ▸).

The aryl-substituted analogue phen­ylglyoxylic acid thio­semicarbazone (H_2_PGTS; benzketozone) has previously been structurally characterized as its free monohydrate (Tillaeva *et al.*, 2022 ▸), but its metal coordination chemistry remains much less explored.
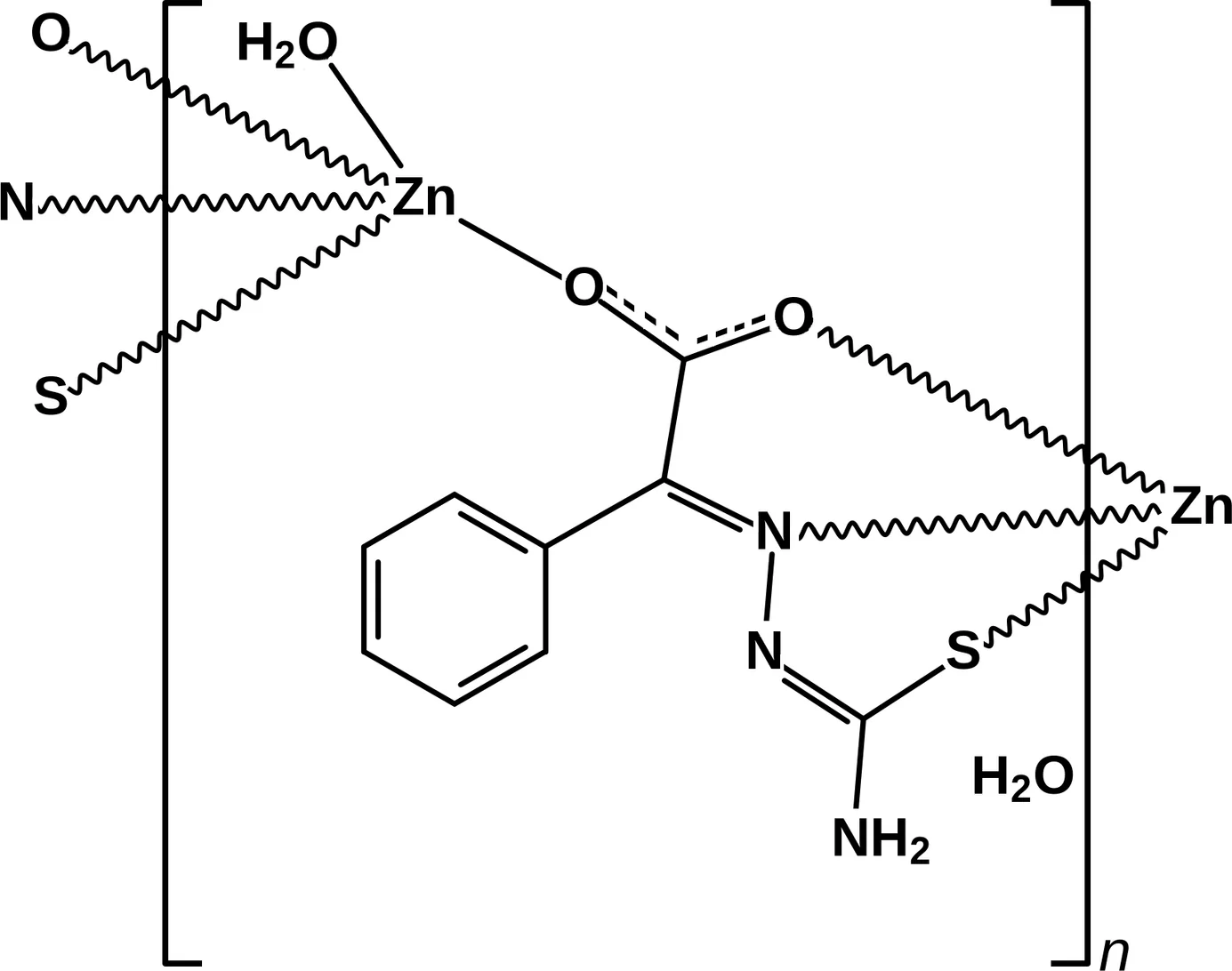


In this context, the title compound, (I), was synthesized and its crystal structure determined by single-crystal X-ray diffraction, with inter­mol­ecular inter­actions qu­anti­fied by Hirshfeld surface analysis.

## 2. Structural commentary

**Figure 1:**
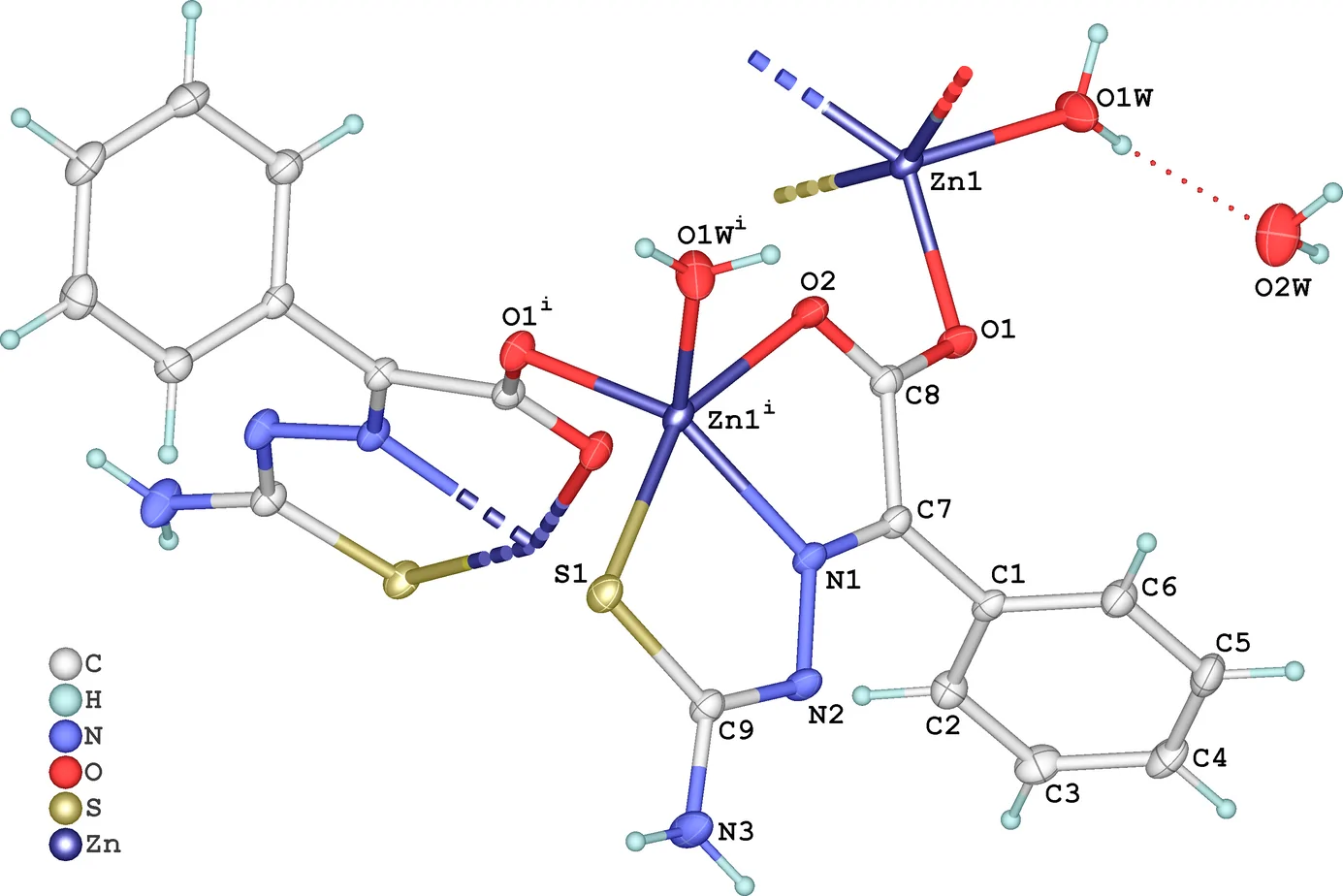
The coordination environment of Zn1 in compound (I), showing the atom-labelling scheme. Displacement ellipsoids are drawn at the 50% probability level, and H atoms are shown as spheres of arbitrary radius. The red dotted line indicates the O—H⋯O hydrogen bond to the non-coordinating water mol­ecule. [Symmetry code: (i) −*x* + 1, *y* − 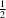, −*z* + 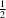.]

The asymmetric unit of (I) contains one Zn^II^ cation, one doubly deprotonated phenyl­glyoxylic acid thio­semicarbazonate ligand, one aqua ligand and one non-coordinating water mol­ecule (Fig. 1 ▸). Zn1 is five-coordinate, bonded to the carboxyl­ate atom O1 and the water O atom O1*W* in the asymmetric unit and to atoms O2^i^, N1^i^ and S1^i^ of a symmetry-related ligand [symmetry code: (i) −*x* + 1, *y* − 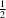, −*z* + 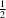]. The coordination environment, with τ_5_ = 0.42 (Addison *et al.*, 1984 ▸), is between the ideal square-pyramidal (*τ*_5_ = 0) and trigonal-bipyramidal (*τ*_5_ = 1) limits, and therefore slightly closer to square-pyramidal. In this description, S1^i^ and O2^i^ define the axial direction [S1^i^—Zn1—O2^i^ = 155.77 (4)°], whereas O1, O1*W* and N1^i^ form the equatorial set of atoms. The three equatorial angles sum up to 358.69 (12)°, and Zn1 is displaced by 0.132 (1) Å from the O1/O1*W*/N1^i^ least-squares plane. The Zn—O distances are 2.0052 (13), 2.0350 (15) and 2.0976 (13) Å, while Zn—N1^i^ and Zn—S1^i^ are 2.1042 (15) and 2.3907 (5) Å, respectively. The two fused five-membered chelate rings, Zn1/N1^i^/N2^i^/C9^i^/S1^i^ and Zn1/N1^i^/C7^i^/C8^i^/O2^i^, are nearly planar, with maximum deviations of 0.053 (2) Å (C9^i^) and 0.074 (1) Å (O2^i^), respectively, and bite angles of 79.98 (4) and 77.11 (5)°.

The ligand is doubly deprotonated, specifically at the carboxyl group and at the hydrazinic atom N2, and coordinates in the thiol­ate form. The C9—S1 distance of 1.7402 (18) Å lies between values expected for localized C=S and C—S bonds. The N2—C9 and N3—C9 distances [1.328 (2) and 1.339 (3) Å] are very similar, indicating substantial delocalization over the thio­amide fragment. The azomethine bond N1=C7 [1.284 (2) Å] and the N1—N2 bond [1.365 (2) Å] are unexceptional. The two carboxyl­ate C—O distances [C8—O1 = 1.270 (2) and C8—O2 = 1.245 (2) Å] are consistent with a deprotonated carboxyl­ate group. The least-squares planes through S1/N1/N2/N3/C1/C7/C9 and O1/O2/C1/C7/C8 are roughly coplanar, forming a dihedral angle of 7.89 (7)°. This is consistent with the torsion angles Zn1—N1^i^—N2^i^—C9^i^ = −3.3 (2)° and N1—C7—C8—O2 = 7.1 (2)°.

The phenyl ring is almost perpendicular to the chelating part of the ligand: it forms dihedral angles of 70.99 (8) and 78.56 (9)° with the thio­semicarbazide (S1/N1/N2/N3/C1/C7/C9) and carboxyl­ate (O1/O2/C1/C7/C8) planes, respectively, and the C2—C1—C7—N1 torsion angle is 71.5 (2)°.

**Figure 2:**
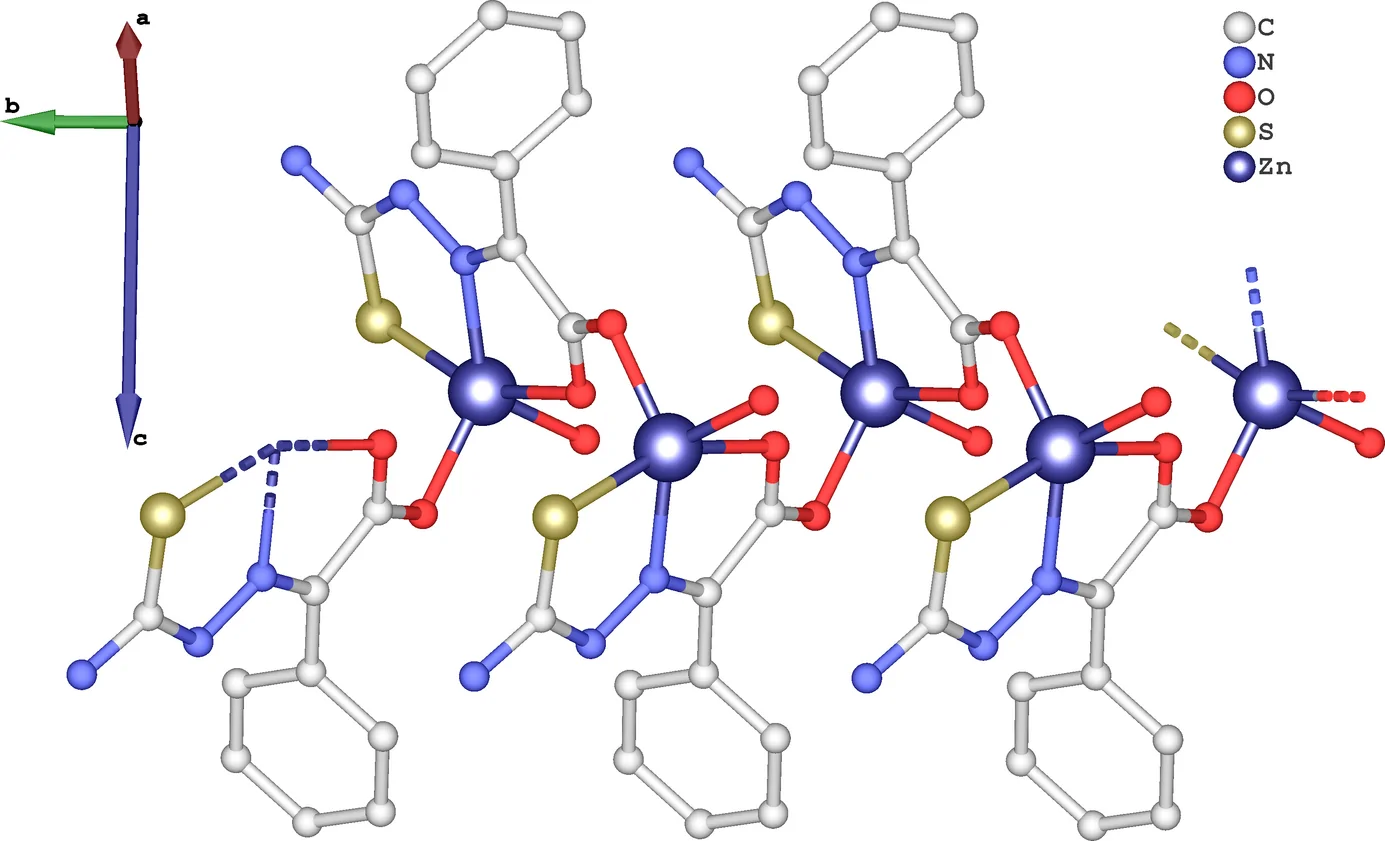
Part of the polymeric chain in (I), propagating parallel to [010] through carboxyl­ate bridges and the tridentate *O*,*N*,*S*-chelation of the ligand. H atoms have been omitted for clarity.

Because the two carboxyl­ate O atoms coordinate to adjacent Zn^II^ cations, each ligand bridges two metal ions and the structure forms a one-periodic coordination polymer. The chain is generated by the 2_1_ screw axis and propagates parallel to [010], with an adjacent Zn1⋯Zn1^i^ separation of 4.8378 (4) Å (Fig. 2 ▸). The bridging carboxyl­ate group adopts a *syn–anti* mode, as shown by the torsion angles Zn1—O1—C8—O2 = −9.7 (2)° and Zn1—O2^i^—C8^i^—O1^i^ = 165.62 (15)°. Consecutive phenyl rings protrude alternately towards opposite sides of the chain.

### 2.1. Supra­molecular features

Hydrogen-bonding involves both water mol­ecules and the terminal amino group N3 (Table 1 ▸). Within each coordination chain, the aqua ligand O1*W* donates H1*WB* to the thiol­ate atom S1 of a screw axis-related repeat unit; this nearly linear O—H⋯S contact is the only conventional hydrogen bond within an individual polymeric chain. The second H atom of the aqua ligand, H1*WA*, is donated to the non-coordinating water mol­ecule O2*W*, which in turn acts as a double donor: H2*WA* is directed to the carboxyl­ate atom O1 of a neighbouring coordination chain, while H2*WB* is donated to a symmetry-related non-coordinating water mol­ecule. Consequently, the non-coordinating water mol­ecules form a hydrogen-bonded chain extending parallel to [010] and simultaneously cross-link neighbouring Zn^II^ coordination chains.

**Figure 3:**
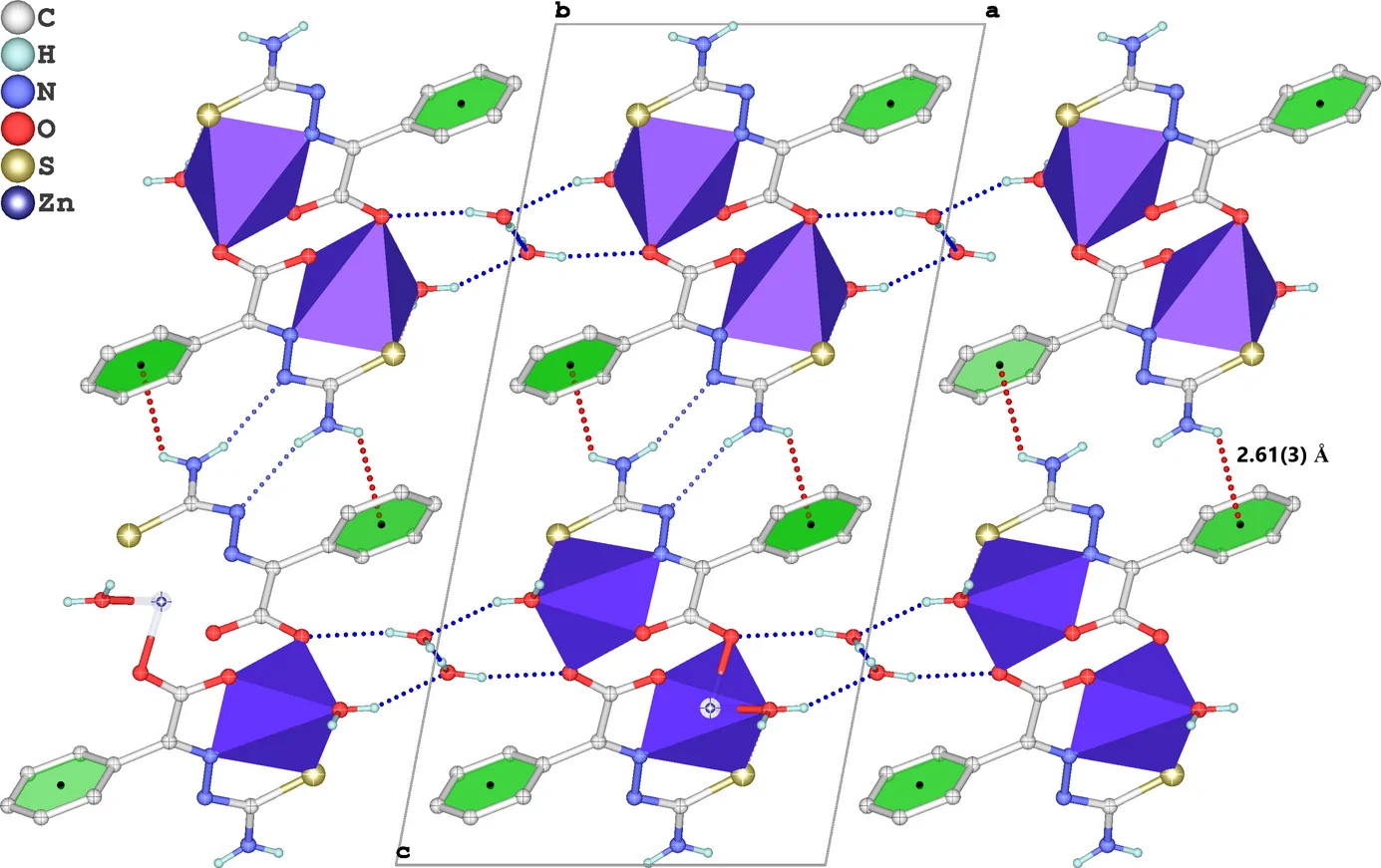
Part of the crystal structure of (I), viewed along the *b* axis, showing the hydrogen bonds (dashed lines) that link neighbouring chains and the non-coordinating water mol­ecules. H atoms not involved in hydrogen bonding have been omitted for clarity.

The terminal amino group N3 provides an additional inter­chain linkage: N3—H3*B*⋯N2 hydrogen bonds between inversion-related chains generate a centrosymmetric 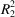
(8) motif (Etter *et al.*, 1990 ▸). The second amino H atom, H3*A*, does not participate in a conventional hydrogen bond but is directed towards the centroid *Cg* of the C1–C6 phenyl ring of the same inversion-related chain, consistent with a weak N—H⋯π contact (Table 1 ▸). No significant π–π stacking is observed between the phenyl rings. The shortest centroid-to-centroid separation, 5.6580 (9) Å, occurs between rings related by translation along the *b* axis; although these rings are parallel, their centroids are laterally offset by 5.019 (2) Å, so that there is no face-to-face overlap. Thus, the supra­molecular assembly is governed primarily by O—H⋯O, O—H⋯S and N—H⋯N hydrogen bonds, assisted by the weak N—H⋯π contact. The intra­chain O—H⋯S inter­actions, together with the water- and amine-mediated inter­chain contacts, link the mono-periodic coordination polymers into a tri-periodic hydrogen-bonded network (Fig. 3 ▸).

## 3. Hirshfeld surface analysis

To qu­antify the inter­molecular contacts contributing to the crystal packing, a Hirshfeld surface analysis (McKinnon *et al.*, 2007 ▸; Spackman & Jayatilaka, 2009 ▸) was performed using *CrystalExplorer* (Spackman *et al.*, 2021 ▸). The surface was generated for the complex part of the asymmetric unit, *i.e.* one [Zn(C_9_H_7_N_3_O_2_S)(H_2_O)] repeat unit of the coordination chain. The non-coordinating water mol­ecule was excluded from this fragment in order to examine its inter­actions with the polymeric chain; however, it was retained as a neighbouring mol­ecule, so that its contacts with the repeat unit, both as a hydrogen-bond donor and as an acceptor, are mapped onto the surface and included in the fingerprint plots. Because the polymer is truncated at the bridging carboxyl­ate group, the Zn⋯O, Zn⋯H and Zn⋯C contacts (6.1% in total) arise mainly from this truncation rather than from genuine inter­molecular packing contacts.

**Figure 4:**
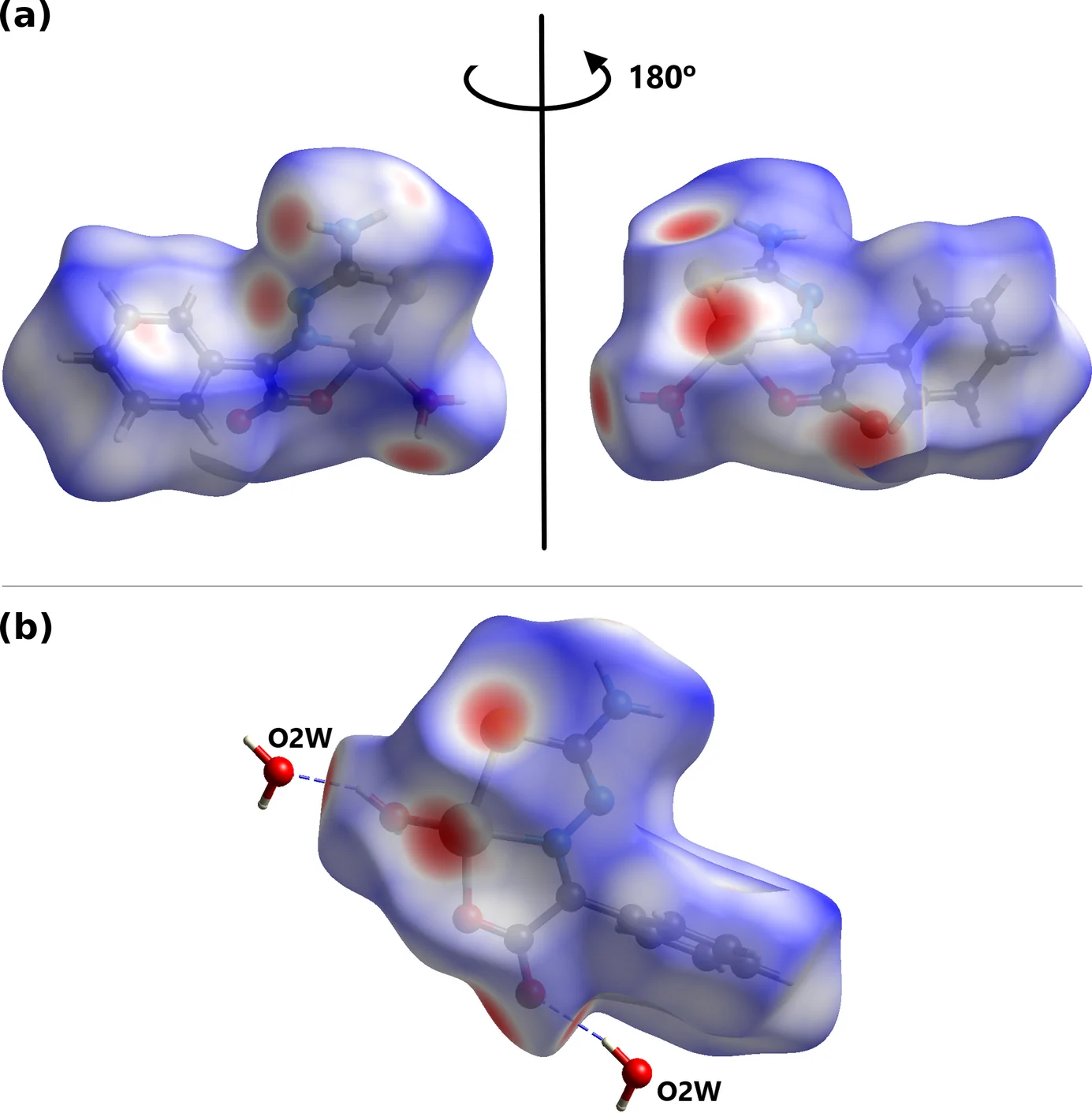
The Hirshfeld surface of (I) mapped over *d*_norm_: (*a*) two views related by a 180° rotation and (*b*) a view showing the O—H⋯O hydrogen bonds (dashed lines) to the neighbouring non-coordinating water mol­ecules (O2*W*).

The Hirshfeld surface mapped over *d*_norm_ is shown in Fig. 4 ▸. The most intense red regions, located near the Zn^II^ cation and the carboxyl­ate O atoms, correspond to the Zn—O coordination bonds to the adjacent units of the chain, which are cut by the truncation of the polymer. The other red spots correspond to the O—H⋯O, O—H⋯S and N—H⋯N hydrogen bonds listed in Table 1 ▸. The contacts with the non-coordinating water mol­ecule are shown explicitly in Fig. 4 ▸*b*: O2*W* accepts a hydrogen bond from the aqua ligand and donates a hydrogen bond to the carboxyl­ate atom O1 of a neighbouring chain, and both inter­actions appear as distinct red spots on the surface. The remaining surface is predominantly white to blue, indicating contacts close to or longer than the sum of the van der Waals radii.

**Figure 5:**
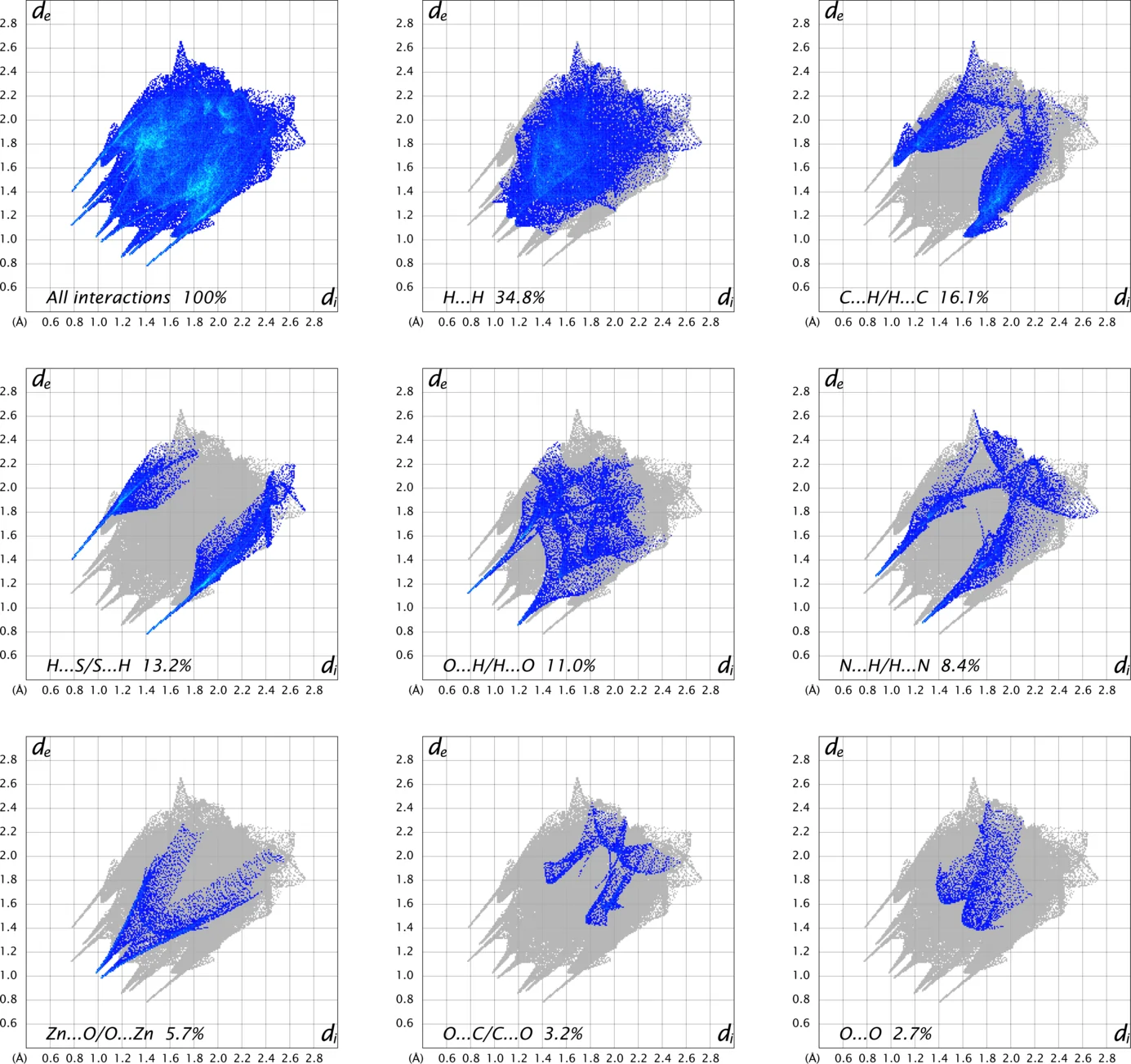
Two-dimensional fingerprint plots for (I), showing the full fingerprint plot and those delineated into H⋯H, C⋯H/H⋯C, H⋯S/S⋯H, O⋯H/H⋯O, N⋯H/H⋯N, Zn⋯O/O⋯Zn, O⋯C/C⋯O and O⋯O contacts, and their relative percentage contributions.

The two-dimensional fingerprint plots (Spackman & McKinnon, 2002 ▸) for the full surface and those delineated into the principal contact types, together with their percentage contributions, are shown in Fig. 5 ▸. H⋯H contacts make the largest contribution, followed by C⋯H/H⋯C, H⋯S/S⋯H, O⋯H/H⋯O and N⋯H/H⋯N contacts; each of the remaining contact types not shown in Fig. 5 ▸ contributes less than 2%. The relatively large H⋯S/S⋯H contribution reflects the exposed thiol­ate S atom, which both coordinates to Zn^II^ and acts as an acceptor in the O—H⋯S hydrogen bond. Overall, the crystal packing is dominated by contacts involving H atoms, consistent with the hydrogen-bonding network described above, while the very small C⋯C contribution (0.6%) confirms the absence of significant π–π stacking.

## 4. Database survey

A search of the Cambridge Structural Database (CSD, Version 6.01, November 2025, including the February 2026 updates; Groom *et al.*, 2016 ▸) was carried out using *ConQuest*, restricted to metal-containing structures and using the α-oxo-carboxyl­ate thio­semicarbazone fragment O—C(O)—C—N—N—C(S)—N with all bond types unspecified. The search returned 97 hits. In most of them, the substituent at the azomethine C atom is H, methyl, ethyl or 2-carb­oxy­ethyl, corresponding to derivatives of glyoxylic, pyruvic, 2-oxobutyric or 2-oxoglutaric acid. The closest aryl-substituted analogue is PIZTOH (Sonawane *et al.*, 1994 ▸), which carries a benzyl substituent. No metal complex with a phenyl group directly bonded to the azomethine C atom was found, so that compound (I) is the first structurally characterized metal complex of this ligand type.

Comparison with the free ligand, H_2_PGTS·H_2_O (GEJFAG; Tillaeva *et al.*, 2022 ▸), shows the changes caused by deprotonation and coordination. In GEJFAG, the ligand is neutral, with protonated carboxyl and hydrazinic N2 groups, and the C9—S1 bond [1.690 (2) Å] corresponds to a thione form. In the crystal structure of (I), C9—S1 is lengthened to 1.7402 (18) Å, consistent with the thiol­ate form. At the same time, N2—C9 is shortened from 1.351 (3) to 1.328 (2) Å and N3—C9 is lengthened from 1.306 (3) to 1.339 (3) Å, so that the two C—N bonds of the thio­amide fragment become nearly equal, indicating charge delocalization after deprotonation of N2. Coordination also changes the ligand conformation considerably. In GEJFAG, the phenyl ring forms dihedral angles of 22.6 (1) and 47.9 (1)° with the thio­semicarbazide (S1/N1/N2/N3/C1/C7/C9) and carboxyl (O1/O2/C1/C7/C8) planes, respectively, and the C2—C1—C7—N1 torsion angle is −27.2 (3)°. In (I), the corresponding values are 70.99 (8), 78.56 (9) and 71.5 (2)°. This rotation is presumably required to relieve steric repulsion between the phenyl ring and the planar, fused O,N,S-chelate system.

Among complexes of the parent glyoxylic acid thio­semicarbazone (H_2_GAT), both discrete and polymeric structures are known. Co^II^ and Ni^II^ form mononuclear octa­hedrally coordinated bis-chelates (ODOWUC and ODOXAJ; Huseynova *et al.*, 2018 ▸). Ni^II^ also forms discrete bis-chelates, [Zn(HGAT)_2_]·*x*H_2_O (FOTPAI, *x* = 5/3; Huseynova *et al.*, 2020 ▸; VADFEP, *x* = 3/2; Huseynova *et al.*, 2022 ▸), in which two monoanionic ligands coordinate to the overall six-coordinate Zn^II^ cation. In FOTPAI, the Zn—O [2.105 (3)–2.197 (3) Å], Zn—N [2.138 (3)–2.175 (3) Å] and Zn—S [2.4974 (12)–2.5874 (13) Å] bonds are all longer than the corresponding bonds in the structure of (I) [2.0052 (13)–2.0976 (13), 2.1042 (15) and 2.3907 (5) Å, respectively], as expected for the lower coordination number and the dianionic ligand in (I).

Only nine of the 97 hits are one-periodic coordination polymers: six Cu, two Pb and one Zn complexes. The Zn^II^ polymer of H_2_GAT (LOXNAQ; Huseynova *et al.*, 2019*b* ▸) is the closest analogue of (I). In both structures, the dianionic ligand chelates one five-coordinate Zn^II^ cation through the O,N,S donor set, a water mol­ecule completes the coordination sphere, and the second carboxyl­ate O atom bridges to the next Zn^II^ ion. Unlike in (I), however, LOXNAQ contains no non-coordinating water mol­ecule, so that the water-mediated cross-linking of the chains observed in (I) is absent. A different mode of chain propagation is found in the Cu^II^ polymer of the same ligand (GERPAU, Burshtein *et al.*, 1987 ▸; redetermined as GERPAU01, Huseynova *et al.*, 2019*a* ▸). Here, the square-pyramidally coordinated Cu^II^ cations are linked through the hydrazinic N atom, and the carboxyl­ate group acts only as a chelating donor. Replacing the H atom of H_2_GAT by a phenyl group in (I) therefore does not change the carboxyl­ate-bridged chain motif. However, the phenyl rings, which protrude alternately to opposite sides of the chain, prevent close packing of the chains and preclude π–π stacking, so that the chains are linked only through hydrogen bonds involving the water mol­ecules and the amino group.

## 5. Synthesis and crystallization

The ligand H_2_PGTS·H_2_O was prepared according to the published procedure (Tillaeva *et al.*, 2022 ▸). A solution of Zn(CH_3_COO)_2_·2H_2_O (0.220 g, 1.00 mmol) in water (5 ml) was added to a solution of H_2_PGTS·H_2_O (0.242 g, 1.00 mmol) in ethanol (5 ml). The reaction mixture was stirred at 333 K at 500 rpm for 10 min and then allowed to evaporate slowly at room temperature. After 5 d, colourless block-shaped crystals suitable for single-crystal X-ray diffraction were obtained (yield 55%). Analysis calculated for C_9_H_11_N_3_O_4_SZn (*M*_r_ = 322.64): C, 33.50; H, 3.44; N, 13.02; S, 9.94%. Found: C, 33.45; H, 3.39; N, 12.98; S, 9.97%.

## 6. Refinement

Crystal data, data collection and structure refinement details are summarized in Table 2 ▸. H atoms attached to C atoms were placed in calculated positions (C—H = 0.93 Å) and refined using a riding model, with *U*_iso_(H) = 1.2*U*_eq_(C). The H atoms of the aqua ligand, the non-coordinating water mol­ecule and of the amino group were located in difference-Fourier maps and refined with restrained O—H and N—H distances of 0.85 (1) and 0.86 (1) Å, respectively. Their positional and isotropic displacement parameters were refined independently. A total of six distance restraints were applied.

## Supplementary Material

Crystal structure: contains datablock(s) I. DOI: 10.1107/S2056989026010169/wm5813sup1.cif

Structure factors: contains datablock(s) I. DOI: 10.1107/S2056989026010169/wm5813Isup2.hkl

CCDC reference: 2591804

Additional supporting information:  crystallographic information; 3D view; checkCIF report

## Figures and Tables

**Table 1:** Hydrogen-bond geometry (Å, °) *Cg* is the centroid of the C1–C6 ring.

*D*—H⋯*A*	*D*—H	H⋯*A*	*D*⋯*A*	*D*—H⋯*A*
O1*W*—H1*WA*⋯O2*W*	0.84 (1)	2.02 (2)	2.809 (3)	155 (3)
O1*W*—H1*WB*⋯S1^i^	0.84 (1)	2.33 (1)	3.1718 (17)	175 (3)
O2*W*—H2*WA*⋯O1^ii^	0.84 (1)	2.19 (1)	3.011 (2)	168 (4)
O2*W*—H2*WB*⋯O2W^ii^	0.84 (1)	2.18 (1)	3.0162 (14)	178 (4)
N3—H3*B*⋯N2^iii^	0.86 (1)	2.28 (1)	3.106 (2)	162 (2)
N3—H3*A*⋯*Cg*^iii^	0.85 (1)	2.61 (3)	3.173 (2)	125 (2)

Symmetry codes: (i) 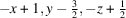; (ii) 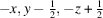; (iii) 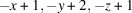.

**Table 2:** Experimental details

Crystal data	
Chemical formula	[Zn(C_9_H_7_N_3_O_2_S)(H_2_O)]·H_2_O
*M*_r_	322.64
Crystal system, space group	Monoclinic, *P*2_1_/*c*
Temperature (K)	291
*a*, *b*, *c* (Å)	10.5337 (1), 5.6580 (1), 21.0089 (3)
β (°)	100.760 (1)
*V* (Å^3^)	1230.11 (3)
*Z*	4
Radiation type	Cu *K*α
μ (mm^−1^)	4.49
Crystal size (mm)	0.3 × 0.2 × 0.12

Data collection	
Diffractometer	Xcalibur, Ruby
Absorption correction	Multi-scan (*CrysAlis PRO*; Rigaku OD, 2023 ▸)
*T*_min_, *T*_max_	0.572, 1.000
No. of measured, independent and observed [*I* > 2σ(*I*)] reflections	7561, 2514, 2354
*R*_int_	0.029
(sin θ/λ)_max_ (Å^−1^)	0.630

Refinement	
*R*[*F*^2^ > 2σ(*F*^2^)], *wR*(*F*^2^), *S*	0.028, 0.078, 1.06
No. of reflections	2514
No. of parameters	188
No. of restraints	6
H-atom treatment	H atoms treated by a mixture of independent and constrained refinement
Δρ_max_, Δρ_min_ (e Å^−3^)	0.29, −0.31

Computer programs: *CrysAlis PRO* (Rigaku OD, 2023 ▸), *SHELXT* (Sheldrick, 2015*a* ▸), *SHELXL* (Sheldrick, 2015*b* ▸), *OLEX2* (Dolomanov *et al.*, 2009 ▸) and *publCIF* (Westrip, 2010 ▸).

## supplementary crystallographic information

### catena-Poly[[[aquazinc(II)]-µ-2-({[amino(sulfanidyl)methylidene]amino}imino)-2-phenylacetato-κ4O,N,S:O'] monohydrate] Crystal data

**Table:**

[Zn(C_9_H_7_N_3_O_2_S)(H_2_O)]·H_2_O	*F*(000) = 656
*M**_r_* = 322.64	*D*_x_ = 1.742 Mg m^−^^3^
Monoclinic, *P*2_1_/*c*	Cu *K*α radiation, λ = 1.54184 Å
*a* = 10.5337 (1) Å	Cell parameters from 5766 reflections
*b* = 5.6580 (1) Å	θ = 4.2–76.1°
*c* = 21.0089 (3) Å	µ = 4.49 mm^−^^1^
β = 100.760 (1)°	*T* = 291 K
*V* = 1230.11 (3) Å^3^	Block, colourless
*Z* = 4	0.3 × 0.2 × 0.12 mm

### catena-Poly[[[aquazinc(II)]-µ-2-({[amino(sulfanidyl)methylidene]amino}imino)-2-phenylacetato-κ4O,N,S:O'] monohydrate] Data collection

**Table:**

Xcalibur, Ruby diffractometer	2514 independent reflections
Radiation source: Enhance (Cu) X-ray Source	2354 reflections with *I* > 2σ(*I*)
Graphite monochromator	*R*_int_ = 0.029
Detector resolution: 10.2576 pixels mm^-1^	θ_max_ = 76.4°, θ_min_ = 4.3°
ω scans	*h* = −13→13
Absorption correction: multi-scan (CrysAlisPro; Rigaku OD, 2023)	*k* = −7→5
*T*_min_ = 0.572, *T*_max_ = 1.000	*l* = −22→26
7561 measured reflections	

### catena-Poly[[[aquazinc(II)]-µ-2-({[amino(sulfanidyl)methylidene]amino}imino)-2-phenylacetato-κ4O,N,S:O'] monohydrate] Refinement

**Table:**

Refinement on *F*^2^	Hydrogen site location: mixed
Least-squares matrix: full	H atoms treated by a mixture of independent and constrained refinement
*R*[*F*^2^ > 2σ(*F*^2^)] = 0.028	*w* = 1/[σ^2^(*F*_o_^2^) + (0.0492*P*)^2^ + 0.2444*P*] where *P* = (*F*_o_^2^ + 2*F*_c_^2^)/3
*wR*(*F*^2^) = 0.078	(Δ/σ)_max_ < 0.001
*S* = 1.06	Δρ_max_ = 0.29 e Å^−^^3^
2514 reflections	Δρ_min_ = −0.31 e Å^−^^3^
188 parameters	Extinction correction: SHELXL2025/1 (Sheldrick 2015b), Fc^*^=kFc[1+0.001xFc^2^λ^3^/sin(2θ)]^-1/4^
6 restraints	Extinction coefficient: 0.0015 (2)

### catena-Poly[[[aquazinc(II)]-µ-2-({[amino(sulfanidyl)methylidene]amino}imino)-2-phenylacetato-κ4O,N,S:O'] monohydrate] Special details

**Table:**

Geometry. All esds (except the esd in the dihedral angle between two l.s. planes) are estimated using the full covariance matrix. The cell esds are taken into account individually in the estimation of esds in distances, angles and torsion angles; correlations between esds in cell parameters are only used when they are defined by crystal symmetry. An approximate (isotropic) treatment of cell esds is used for estimating esds involving l.s. planes.

### catena-Poly[[[aquazinc(II)]-µ-2-({[amino(sulfanidyl)methylidene]amino}imino)-2-phenylacetato-κ4O,N,S:O'] monohydrate] Fractional atomic coordinates and isotropic or equivalent isotropic displacement parameters (Å^2^)

**Table:**

	*x*	*y*	*z*	*U*_iso_*/*U*_eq_	
Zn1	0.33743 (2)	0.15367 (4)	0.18671 (2)	0.02765 (12)	
S1	0.76756 (4)	0.93004 (9)	0.39192 (2)	0.03347 (14)	
O1	0.32015 (13)	0.3021 (2)	0.27135 (6)	0.0330 (3)	
O1W	0.19866 (16)	−0.0979 (3)	0.18463 (10)	0.0505 (4)	
H1WA	0.1198 (13)	−0.091 (6)	0.1870 (17)	0.074 (10)*	
H1WB	0.210 (3)	−0.227 (3)	0.1666 (15)	0.073 (10)*	
O2	0.52699 (13)	0.3937 (2)	0.27595 (7)	0.0339 (3)	
N1	0.51936 (14)	0.6934 (3)	0.37017 (7)	0.0271 (3)	
N2	0.52557 (16)	0.8524 (3)	0.41950 (8)	0.0324 (4)	
N3	0.64759 (19)	1.1381 (4)	0.47600 (10)	0.0444 (5)	
C1	0.29820 (16)	0.5810 (3)	0.38054 (8)	0.0271 (4)	
C2	0.21642 (19)	0.7747 (4)	0.36741 (9)	0.0340 (4)	
H2	0.238097	0.898891	0.342474	0.041*	
C3	0.1029 (2)	0.7831 (4)	0.39137 (11)	0.0410 (5)	
H3	0.047601	0.911604	0.381886	0.049*	
C4	0.0713 (2)	0.6005 (4)	0.42947 (10)	0.0413 (5)	
H4	−0.004767	0.607332	0.445818	0.050*	
C5	0.1526 (2)	0.4085 (4)	0.44320 (10)	0.0417 (5)	
H5	0.131217	0.286301	0.468878	0.050*	
C6	0.2663 (2)	0.3973 (4)	0.41872 (10)	0.0349 (4)	
H6	0.320883	0.267589	0.427841	0.042*	
C7	0.41709 (17)	0.5678 (3)	0.35307 (8)	0.0267 (4)	
C8	0.42359 (17)	0.4086 (3)	0.29632 (8)	0.0274 (4)	
C9	0.63595 (17)	0.9720 (3)	0.42991 (8)	0.0300 (4)	
O2W	−0.03563 (18)	−0.1447 (4)	0.22900 (11)	0.0613 (5)	
H2WA	−0.1165 (10)	−0.148 (6)	0.2234 (19)	0.081 (11)*	
H2WB	−0.016 (4)	−0.283 (3)	0.2415 (19)	0.088 (12)*	
H3A	0.7214 (14)	1.202 (4)	0.4853 (13)	0.044 (7)*	
H3B	0.5869 (19)	1.153 (4)	0.4977 (11)	0.046 (7)*	

### catena-Poly[[[aquazinc(II)]-µ-2-({[amino(sulfanidyl)methylidene]amino}imino)-2-phenylacetato-κ4O,N,S:O'] monohydrate] Atomic displacement parameters (Å^2^)

**Table:**

	*U*^11^	*U*^22^	*U*^33^	*U*^12^	*U*^13^	*U*^23^
Zn1	0.02673 (16)	0.03423 (18)	0.02422 (16)	−0.00090 (9)	0.01046 (10)	−0.00270 (9)
S1	0.0263 (2)	0.0425 (3)	0.0332 (2)	−0.00429 (17)	0.00958 (17)	−0.00229 (18)
O1	0.0305 (7)	0.0431 (7)	0.0263 (6)	−0.0046 (5)	0.0078 (5)	−0.0097 (5)
O1W	0.0379 (8)	0.0392 (8)	0.0795 (12)	−0.0112 (7)	0.0243 (8)	−0.0157 (8)
O2	0.0345 (7)	0.0385 (7)	0.0324 (7)	−0.0053 (6)	0.0155 (5)	−0.0094 (6)
N1	0.0258 (7)	0.0354 (8)	0.0211 (7)	−0.0024 (6)	0.0070 (5)	−0.0035 (6)
N2	0.0296 (8)	0.0430 (9)	0.0260 (7)	−0.0071 (6)	0.0091 (6)	−0.0111 (6)
N3	0.0385 (10)	0.0562 (12)	0.0412 (10)	−0.0172 (8)	0.0145 (8)	−0.0212 (9)
C1	0.0244 (8)	0.0367 (9)	0.0208 (7)	−0.0034 (7)	0.0058 (6)	−0.0052 (7)
C2	0.0327 (9)	0.0378 (10)	0.0319 (9)	0.0002 (8)	0.0071 (7)	0.0019 (8)
C3	0.0300 (10)	0.0527 (12)	0.0403 (11)	0.0087 (9)	0.0066 (8)	−0.0028 (9)
C4	0.0290 (9)	0.0630 (13)	0.0349 (10)	−0.0016 (9)	0.0137 (8)	−0.0040 (9)
C5	0.0421 (11)	0.0508 (12)	0.0364 (10)	−0.0075 (9)	0.0181 (9)	0.0036 (9)
C6	0.0357 (10)	0.0388 (10)	0.0324 (9)	0.0021 (8)	0.0115 (8)	0.0030 (8)
C7	0.0261 (8)	0.0335 (9)	0.0212 (8)	−0.0021 (7)	0.0064 (6)	−0.0014 (7)
C8	0.0307 (9)	0.0309 (9)	0.0215 (8)	−0.0005 (7)	0.0069 (6)	−0.0004 (7)
C9	0.0289 (8)	0.0377 (10)	0.0237 (8)	−0.0021 (7)	0.0060 (6)	−0.0012 (7)
O2W	0.0398 (10)	0.0780 (14)	0.0641 (12)	−0.0045 (9)	0.0040 (8)	0.0210 (10)

### catena-Poly[[[aquazinc(II)]-µ-2-({[amino(sulfanidyl)methylidene]amino}imino)-2-phenylacetato-κ4O,N,S:O'] monohydrate] Geometric parameters (Å, º)

**Table:**

Zn1—S1^i^	2.3907 (5)	C1—C2	1.389 (3)
Zn1—O1	2.0052 (13)	C1—C6	1.392 (3)
Zn1—O1W	2.0350 (15)	C1—C7	1.475 (2)
Zn1—O2^i^	2.0976 (13)	C2—H2	0.9300
Zn1—N1^i^	2.1042 (15)	C2—C3	1.382 (3)
S1—C9	1.7402 (18)	C3—H3	0.9300
O1—C8	1.270 (2)	C3—C4	1.385 (3)
O1W—H1WA	0.843 (10)	C4—H4	0.9300
O1W—H1WB	0.844 (10)	C4—C5	1.380 (3)
O2—C8	1.245 (2)	C5—H5	0.9300
N1—N2	1.365 (2)	C5—C6	1.391 (3)
N1—C7	1.284 (2)	C6—H6	0.9300
N2—C9	1.328 (2)	C7—C8	1.506 (2)
N3—C9	1.339 (3)	O2W—H2WA	0.838 (10)
N3—H3A	0.847 (10)	O2W—H2WB	0.840 (10)
N3—H3B	0.856 (10)		
			
O1—Zn1—S1^i^	103.41 (4)	C1—C2—H2	120.0
O1—Zn1—O1W	97.37 (7)	C3—C2—C1	120.07 (19)
O1—Zn1—O2^i^	97.49 (6)	C3—C2—H2	120.0
O1—Zn1—N1^i^	130.72 (6)	C2—C3—H3	119.9
O1W—Zn1—S1^i^	101.83 (6)	C2—C3—C4	120.2 (2)
O1W—Zn1—O2^i^	87.34 (7)	C4—C3—H3	119.9
O1W—Zn1—N1^i^	130.60 (7)	C3—C4—H4	119.9
O2^i^—Zn1—S1^i^	155.77 (4)	C5—C4—C3	120.11 (19)
O2^i^—Zn1—N1^i^	77.11 (5)	C5—C4—H4	119.9
N1^i^—Zn1—S1^i^	79.98 (4)	C4—C5—H5	119.9
C9—S1—Zn1^ii^	95.78 (6)	C4—C5—C6	120.1 (2)
C8—O1—Zn1	110.63 (11)	C6—C5—H5	119.9
Zn1—O1W—H1WA	133 (2)	C1—C6—H6	120.1
Zn1—O1W—H1WB	117 (2)	C5—C6—C1	119.77 (19)
H1WA—O1W—H1WB	107 (3)	C5—C6—H6	120.1
C8—O2—Zn1^ii^	113.71 (11)	N1—C7—C1	126.26 (16)
N2—N1—Zn1^ii^	124.33 (11)	N1—C7—C8	113.11 (15)
C7—N1—Zn1^ii^	115.78 (12)	C1—C7—C8	120.49 (15)
C7—N1—N2	119.72 (15)	O1—C8—C7	116.67 (15)
C9—N2—N1	112.18 (15)	O2—C8—O1	124.32 (16)
C9—N3—H3A	115.1 (18)	O2—C8—C7	118.99 (16)
C9—N3—H3B	118.7 (18)	N2—C9—S1	127.08 (14)
H3A—N3—H3B	125 (3)	N2—C9—N3	115.87 (17)
C2—C1—C6	119.74 (17)	N3—C9—S1	117.02 (14)
C2—C1—C7	120.16 (17)	H2WA—O2W—H2WB	102 (3)
C6—C1—C7	120.10 (17)		
			
Zn1^ii^—S1—C9—N2	8.01 (18)	C1—C2—C3—C4	1.1 (3)
Zn1^ii^—S1—C9—N3	−174.01 (16)	C1—C7—C8—O1	4.7 (3)
Zn1—O1—C8—O2	−9.7 (2)	C1—C7—C8—O2	−176.99 (17)
Zn1—O1—C8—C7	168.47 (12)	C2—C1—C6—C5	0.3 (3)
Zn1^ii^—O2—C8—O1	165.62 (15)	C2—C1—C7—N1	71.5 (2)
Zn1^ii^—O2—C8—C7	−12.5 (2)	C2—C1—C7—C8	−103.8 (2)
Zn1^ii^—N1—N2—C9	−3.3 (2)	C2—C3—C4—C5	−0.6 (3)
Zn1^ii^—N1—C7—C1	−173.36 (14)	C3—C4—C5—C6	−0.1 (3)
Zn1^ii^—N1—C7—C8	2.2 (2)	C4—C5—C6—C1	0.3 (3)
N1—N2—C9—S1	−4.4 (2)	C6—C1—C2—C3	−1.0 (3)
N1—N2—C9—N3	177.57 (17)	C6—C1—C7—N1	−109.7 (2)
N1—C7—C8—O1	−171.16 (16)	C6—C1—C7—C8	75.0 (2)
N1—C7—C8—O2	7.1 (2)	C7—N1—N2—C9	−178.26 (17)
N2—N1—C7—C1	2.1 (3)	C7—C1—C2—C3	177.80 (18)
N2—N1—C7—C8	177.66 (15)	C7—C1—C6—C5	−178.50 (17)

Symmetry codes: (i) −*x*+1, *y*−1/2, −*z*+1/2; (ii) −*x*+1, *y*+1/2, −*z*+1/2.

### catena-Poly[[[aquazinc(II)]-µ-2-({[amino(sulfanidyl)methylidene]amino}imino)-2-phenylacetato-κ4O,N,S:O'] monohydrate] Hydrogen-bond geometry (Å, º)

*Cg* is the centroid of the C1–C6 ring.

**Table:**

*D*—H···*A*	*D*—H	H···*A*	*D*···*A*	*D*—H···*A*
O1*W*—H1*WA*···O2*W*	0.84 (1)	2.02 (2)	2.809 (3)	155 (3)
O1*W*—H1*WB*···S1^iii^	0.84 (1)	2.33 (1)	3.1718 (17)	175 (3)
O2*W*—H2*WA*···O1^iv^	0.84 (1)	2.19 (1)	3.011 (2)	168 (4)
O2*W*—H2*WB*···O2W^iv^	0.84 (1)	2.18 (1)	3.0162 (14)	178 (4)
N3—H3*B*···N2^v^	0.86 (1)	2.28 (1)	3.106 (2)	162 (2)
N3—H3*A*···Cg^v^	0.85 (1)	2.61 (3)	3.173 (2)	125 (2)

Symmetry codes: (iii) −*x*+1, *y*−3/2, −*z*+1/2; (iv) −*x*, *y*−1/2, −*z*+1/2; (v) −*x*+1, −*y*+2, −*z*+1.
